# The Effects of FSH Versus GnRH Vaccination on Growth Performance and Meat Quality of Surgically Castrated Male Growing-Finishing Pigs

**DOI:** 10.3390/ani15213134

**Published:** 2025-10-29

**Authors:** Ganchuan Wang, Junhua Zhou, Gang Lv, Xuemei Jiang, Chenling Song, Lun Hua, Chunxi Wang, Chao Jin, De Wu, Xingfa Han, Yong Zhuo

**Affiliations:** 1 Animal Nutrition Institute, Sichuan Agricultural University, Chengdu 611130, China; 2023314118@stu.sicau.edu.cn (G.W.); lvgang@feederbio.com (G.L.); 71310@sicau.edu.cn (X.J.); 14084@sicau.edu.cn (C.S.); hualun@sicau.edu.cn (L.H.); jinchao@sicau.edu.cn (C.J.); wude@sicau.edu.cn (D.W.); 2 School of Life Sciences, Sichuan Agricultural University, Yaan 625014, China; lihaidebabayyds@gmail.com; 3 Research and Development Department, Feeder Biotechnology (Sichuan) Co., Ltd., Chengdu 610041, China; 4 Kunming Saturn Biological Technology (Group) Co., Ltd., Kunming 650217, China; wcx@saturn-bio.com

**Keywords:** follicle-stimulating hormone, gonadotropin-releasing hormone, growth performance, meat quality, pigs

## Abstract

**Simple Summary:**

This study evaluated the effects of follicle-stimulating hormone vaccination following surgical castration on the growth performance, carcass traits, and meat quality of growing-finishing pigs. A 12-week trial involving 24 finishing pigs demonstrated that the FSH group exhibited a significant improvement in feed efficiency three weeks after booster vaccination compared to the GnRH-immunized group. Additionally, a reduction in average backfat thickness, abdominal fat weight, and abdominal fat percentage was observed in the FSH-immunized group, along with a significant increase in the liver index compared to the Placebo-immunized group. Collectively follicle-stimulating hormone vaccination can effectively improve feed efficiency and reduce adipose tissue deposition in castrated male pigs to a certain extent. Additionally, the meat quality traits were also impacted by the follicle-stimulating hormone vaccination.

**Abstract:**

Previous studies have demonstrated that follicle-stimulating hormone (FSH) plays a critical role in promoting adipogenesis. Surgical castration results in elevated FSH concentrations in pigs, and is accompanied by reduced feed efficiency. This study aimed to investigate the effects of a novel FSH vaccine comprising FSHβ13AA-tandem-ovalbumin conjugate on growth performance and meat quality in barrows. Twenty-four barrows with initial body weight of 32.54 ± 1.90 kg and 12-week age, were randomly assigned to three groups: Placebo-immunized, GnRH-immunized, and FSH-immunized. At three weeks after booster immunization, the FSH-immunized group exhibited significantly improved feed efficiency compared to GnRH-immunized barrows. Compared to the Placebo-immunized group, GnRH and FSH vaccination reduced average backfat thickness by 0.90% and 4.55%, abdominal fat weight by 3.49% and 10.56%, and abdominal fat percentage by 4.09% and 11.95%, respectively. Moreover, the FSH-immunized group showed a significant increase in liver weight relative to the Placebo-immunized group. In addition, compared with the control group, at slaughter FSH vaccination significantly increased muscle pH at 45 min, drip loss at 24 h, and shear force (*p* < 0.05). These results demonstrate that active immunization against FSH in barrows can enhance feed efficiency and reduce adiposity, as well as influence the meat quality.

## 1. Introduction

Pork constitutes a primary source of animal protein and essential fatty acids for humans. However, pork quality can be significantly affected by several factors, with particular concern arising from the accumulation of androstenone and skatole in adipose tissues. These bioactive compounds, produced specifically in sexually mature uncastrated boars, generate a distinctive malodor reminiscent of urine and feces, clinically termed ‘boar taint’ [1]. To address this issue, surgical castration has been extensively implemented in global swine production systems. Beyond mitigating boar taint, this practice effectively reduces aggressive behaviors including fighting and mounting [2,3,4]. However, castration also induces significant physiological alterations in porcine lipid metabolism, manifesting as: (1) increased carcass fat deposition (typically 5% greater separable fat content compared to intact boars); (2) modified fatty acid profiles characterized by reduced backfat protein content, elevated lipid content, and diminished unsaturated fatty acid concentrations [3,4,5] (3) compromised feed efficiency demonstrated by substantially higher feed intake and reduced feed conversion ratios relative to intact males [6,7,8].

In swine production systems, surgical castration of piglets at a young age leads to a sustained elevation in circulating FSH levels [9,10] and increased carcass fat accretion compared to intact boars [3,9,10,11,12]. In contrast, boars immunized with GnRH showed reduced circulating FSH levels and lower carcass fat content [3,10,11,12]. Therefore, FSH has been considered as an important regulator of fat accumulation in barrows [13,14,15]. Mechanically, the increase in FSH levels post-castration stimulates lipogenesis and fat deposition in male pigs by activating the PPARγ-mediated lipogenic signaling pathway in adipose tissue [16].

We previously developed a novel follicle-stimulating hormone vaccine using FSHβ-13AA tandem peptides conjugated to ovalbumin. When formulated with the mild adjuvant specol, this vaccine could significantly reduce weight gain and prevent ovariectomy-induced obesity in mice. Additionally, it also could effectively suppress adipose tissue accumulation in both intact male and female mice [17]. Fat accumulation is tightly and negatively correlated to feed efficiency. Therefore, we speculate that the increased secretion of FSH following surgical castration may also be a significant factor contributing to the reduced feed efficiency in barrows.

However, to date, there are no studies investigating the causative link between increased FSH secretion and reduced feed efficiency in borrows. This study was therefore aimed to test whether vaccination with our novel FSH vaccine can improve feed efficiency in castrated male pigs. Intriguingly, we firstly found that FSH vaccination could efficiently improve feed efficiency of castrated male pigs, possibly by enlarging liver and reducing adipose accumulation. Our novel findings establish a theoretical and technical foundation for the potential application of FSH vaccination as an effective strategy to improve feed efficiency in swine production.

## 2. Materials and Methods

### 2.1. Experimental Design and Animal Feeding Management

Twenty-four nursery pigs (DLY breed, a cross of Duroc, Landrace, and Large breeds) with an average weight of 32.54 ± 1.90 kg and 12-week age, were randomly assigned into three treatment groups. All pigs, procured from Decon Agriculture and Animal Husbandry Food Group Co., Ltd. (Chengdu, China), were handled in accordance with the guidelines approved by the Animal Care and Use Committee of Sichuan Agricultural University (Approval No. SICAU—20220106 on 3 March 2022). And all pigs were castrated at the age of 7 days. Each treatment consisted of 8 replicates, with one pig per replicate. Three groups were: (1) Placebo-immunized, which received an emulsifier only without any vaccine; (2) GnRH-immunized, and (3) FSH-immunized. The experimental duration spanned 13 weeks.

The experiment utilized a corn-soybean meal-based basal diet formulated according to the nutritional guidelines specified in Nutrient Requirements of Swine (NRC, 2012) [18]. All dietary formulations (Table 1) were processed at the Animal Nutrition Research Facility of Sichuan Agricultural University. Notably, the dietary formulations for each growth stage in the present study incorporated the amino acid requirements established by NRC (2012) [18] for intact boars. It is generally accepted that intact boars exhibit substantially greater amino acid requirements compared to castrated barrows, which aligns with NRC recommendations differentiating nutrient specifications based on sex and physiological status.

Prior to the experiment, the pens underwent thorough washing and fumigation with paraformaldehyde combined with potassium permanganate for 48 h. Subsequently, the windows were opened for ventilation for two days. Following this, the pens were sprayed with sodium hydroxide for 12 h and then washed twice before allowing the pigs to enter. The pigs were housed in one pen and individually fed with free access to feed. Throughout the experiment, the piglets were fed twice daily at 09:00 and 15:00, with ad libitum access to water. Daily monitoring of the health status and food consumption of the piglets was conducted. The condition of the piglets was accurately recorded daily, while their health status was regularly observed. The feed tanks were cleaned every two days, remaining feed was recorded, and feed intake was documented. All animal procedures adhered to the guidelines outlined by the Animal Care and Use Committee of Sichuan Agricultural University (Approval No. SICAU—20220106 on 3 March 2022).

### 2.2. Vaccine Preparation and Administration

At the initiation of the experiment, castrated boars received an intramuscular injection in the cervical region to establish primary immunization, with both the GnRH vaccine group and FSH vaccine group being administered 2.0 mL of corresponding vaccines. Pigs in GnRH and FSH vaccine groups were inoculated with a conjugate containing 100 μg of GnRH or FSH peptide equivalent, respectively. A booster immunization was performed at week 3 using identical dosages. The control group was injected with the emulsion adjuvant only.

The vaccine was prepared as described by Oonk et al. (1998) [19]. Specifically as follows: A tandem dimeric GnRH peptide was synthesized by substituting the glycine residue at position 6 of the decapeptide (TDK) with D-lysine, then conjugated to ovalbumin (OVA) as the carrier protein. The conjugate was emulsified in Specol adjuvant (ID-Lelystad formulation). The FSH vaccine was prepared by dissolving the FSHβ-13AA-T-OVA conjugate in 0.85% (*w*/*v*) physiological saline to form the aqueous phase. This aqueous phase (45% *v*/*v*) was mixed with the oil phase (Specol adjuvant, 55% *v*/*v*) at a 4:5 ratio. The mixture was homogenized using an Ultraturrax homogenizer (IKA, Staufen, Germany) at 8000 rpm under continuous stirring until a stable water-in-oil emulsion with homogeneous consistency was achieved [10,20,21].

### 2.3. Sample Collections

Blood samples were collected from each pig at 3-week intervals during the experiment. Prior to blood collection, the pigs were fasted for 12 h but allowed free access to water. Approximately 10 mL of venous blood was drawn from the anterior vena cava using a sterile syringe and immediately transferred into vacuum blood collection tubes. The blood samples were kept at room temperature in a horizontal position for 30 min to allow clotting, followed by centrifugation at 3000 rpm for 15 min at 4 °C. The serum was then separated, and the supernatant was aliquoted into 1.5 mL microcentrifuge tubes and stored at −20 °C for further analysis.

Six pigs per treatment group were randomly selected for slaughter at 9 weeks post-booster vaccination. Following euthanasia, pig abdominal cavities were immediately dissected. Internal organs were aseptically separated and weighed, followed by measurement of backfat thickness at the dorsal midline: the thickest part over the shoulders (near the first thoracic vertebra, T1), the thoracolumbar junction (between the last thoracic and first lumbar vertebra, T-L), and the lumbosacral junction (posterior to the sixth lumbar vertebra, L6) and then give an average.

### 2.4. Determination of Growth Performance

On days 1, 21, 42, 63, and 84 of the experiment (representing Weeks 1, 3, 6, 9, and 12), all pigs were fasted and weighed at 8:00 a.m. to determine weekly and overall average daily gain (ADG). Feed intake was recorded daily, and average daily feed intake (ADFI) and feed conversion ratio (FCR) were calculated as follows:

ADFI = (Total feed offered − residual feed − wasted feed)/Number of experimental days

ADG = (Final body weight − initial body weight)/Number of Experimental Days

FCR = ADFI/ADG

### 2.5. Serum Biochemical Indicators

Serum lipid profiles and metabolic parameters, including total cholesterol (TC), high-density lipoprotein cholesterol (HDL-C), glucose (GLU), and urea (UREA), were quantitatively analyzed using an automated biochemistry analyzer (Hitachi 3100, Japan).

### 2.6. Meat Quality Determination

Meat quality parameters were assessed on the left carcass side as follows:

Meat color: At 45 min post-slaughter, colorimetric parameters of longissimus dorsi muscle samples were measured using a chroma meter (colorimeter). Three replicate measurements per sample were recorded for redness (a*45 min), yellowness (b*45 min), and lightness (L*45 min). Samples were placed in individually labeled bags (e.g., “meat color 1-1”) with paper tags and stored at 4 °C for 24 h. After refrigeration, the colorimetric analysis procedures for a*24 h, b*24 h, and L*24 h were repeated using the identical methodology as above [22].

pH Measurement: At 45 min post-slaughter, the pH meter probe was inserted into the longissimus dorsi muscle sample to a depth >1.5 cm. The pH value (designated as pH45) was recorded after stabilization. Triplicate measurements were performed for each sample. Subsequently, the muscle sample was placed in a labeled bag and stored at 4 °C. After 24 h of refrigerated storage, the pH was measured again (designated as pH24) [22].

Drip loss measurement: Drip loss was typically determined at 24 h and 48 h post-slaughter. Fresh meat samples (2.5 cm thick striploins) were weighed initially. One end of each sample was hooked with stainless steel wire and suspended inside a zip-lock bag (ensuring no contact between the meat and bag). The bag was sealed and stored at 4 °C. After 24 h, the sample was removed, surface moisture was blotted using filter paper, and the sample was reweighed to calculate 24 h drip loss. The same sample was then re-hung for an additional 24 h (total 48 h) to determine cumulative 48 h drip loss [22].

Cooking loss: Fresh muscle samples (approximately 100 g) were weighed 45 min after slaughter and placed into a boiling water bath for cooking for 30 min. The cooked muscle was taken out and hung at room temperature for 20 min, then reweighed to calculate cooking loss for 45 min. Additionally, longissimus dorsi muscle samples (approximately 100 g) were placed at 4 °C for 24 h to determine cooking loss for 24 h [23].

Shear force: Longissimus dorsi muscle samples were collected and stored at 4 °C for 24 h prior to shear force analysis. Uniform meat pieces (approximately 6 cm in length, width, and height) were trimmed and placed in self-sealing bags. A thermometer was inserted into the geometric center of each sample. Samples were heated in a water bath until the core temperature reached 75 °C. After heating, samples were immediately cooled at 4 °C. Cylindrical cores (1.27 cm diameter) were extracted from each sample using a circular coring device. Four technical replicates per sample were prepared by trimming excess tissue. Shear force was measured using a texture analyzer [24].

Marbling Score Assessment: After 24 h of refrigeration at 4 °C, muscle samples were extracted. A fresh transverse section of the muscle was made using a scalpel or sharp blade, and the marbling score was evaluated by visual comparison with a standardized marbling score chart. To ensure accuracy, 3–4 independent evaluators were involved in the scoring process, with an allowable inter-rater deviation of approximately ± 0.5 points [22].

Intramuscular fat (IMF) determination: The Soxhlet extraction method was employed. After freeze-drying and homogenizing muscle samples, lipids were extracted using an organic solvent (e.g., diethyl ether or petroleum ether) under reflux conditions. The fat content was then calculated as a percentage of the dry matter weight [25,26].

### 2.7. Antibody Titer Analysis

Both serum circulating anti-FSH and anti-GnRH antibody titers were quantified using an enzyme-linked immunosorbent assay (ELISA). Ninety-six-well microplates (Thermo Fisher Scientific, Waltham, MA, USA) were coated overnight at 4 °C with FSH β-subunit 13AA-T peptide or G6K-GnRH tandem peptide. Following three washes with PBS containing 0.05% Tween-20 (PBST), nonspecific binding sites were blocked with 300 μL of 5% (*w*/*v*) skim milk (Molico, Nestlé, Araçatuba, Brazil) in PBST for 30 min at 37 °C. After additional PBST washes, 50 μL of serum at 1:1000 dilution was added and incubated at 37 °C for 1 h. Each plate included two negative controls: PBST alone and pre-immunization porcine serum. Plates were washed again before adding 100 μL of horseradish peroxidase (HRP)-conjugated goat anti-swine IgG (Sigma-Aldrich, Burlington, MA, USA; 1:30,000 dilution in PBST) and incubating for 1 h at 37 °C. Following three final washes, the reaction was developed with 3,3’,5,5’-tetramethylbenzidine (TMB; Sigma-Aldrich) for 15 min at room temperature, then stopped with 2M sulfuric acid. Absorbance was measured at 450 nm using a microplate reader (Tecan Sunrise, Männedorf, Switzerland) [17].

### 2.8. Statistical Analysis

Experimental data were analyzed with the Statistical Analysis System, Version 9.2 (SAS institute, Cary, NC, USA). The effects of treatment group on serum antibody titters and metabolic parameters were evaluated with a Mixed-Models ANOVA. The model included fixed effects of treatment, sampling occasion, and their interaction, and random effects of pig within treatment. Since serum analyses were performed repeatedly, sampling occasion was analyzed with a repeated statement, using various alternatives for the covariance structure (the model with the smallest AKaike’s Information Criterion values was chosen). For both serum anti-GnRH and anti-FSH antibody titers, the covariance structure was the Autoregressive of order 1; and for serum TC, glucose, urea and HDL-C, it was Variance Components. All other remaining parameters were analyzed by one-way ANOVA using the General Linear Models (GLM) procedure. When applicable, multiple comparisons were made using Duncan’s method. All data was undergone normality test using PROC UNIVARIATE with the NORMAL Statement, before statistical analysis. Both serum anti-GnRH and anti-FSH antibody titers were log-transformed to normalize their distribution. Back-transformed means without correction were reported. Differences were considered statistically significant at *p* < 0.05, while 0.05 ≤ *p* ≤ 0.10 was interpreted as a trend. All data are presented as mean ± standard deviation (SD).

## 3. Results

### 3.1. Changes in Serum Antibody Titers for FSH or GnRH Vaccination

**Figure 1:**
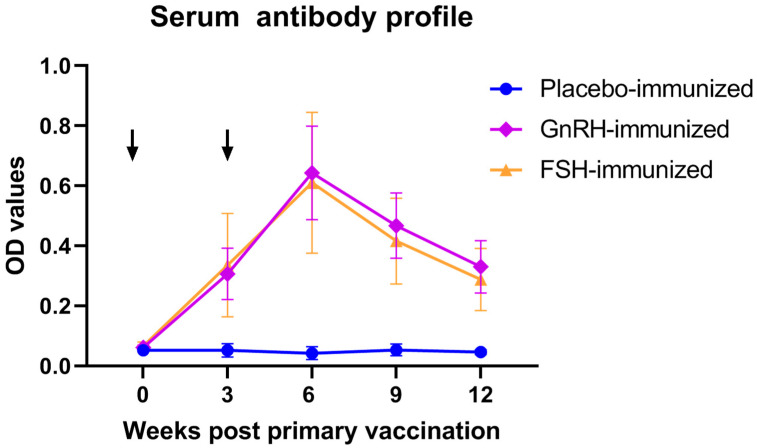
Serum antibody profile following anti-GnRH or anti-FSH vaccination. Arrows indicate primary vaccination and subsequent booster at week 3.

Serum antibody titers against FSH or GnRH were longitudinally monitored throughout the feeding trial. Notably, immunization of barrows with either FSH or GnRH both induced a robust humoral immune response (Figure 1) at weeks 3, 6, 9, and 12 (*p* < 0.05), confirming successful vaccine seroconversion.

### 3.2. The Impact of FSH or GnRH Vaccination on Growth Performance

We found that neither GnRH vaccination nor FSH vaccination had a significant effect on the ADFI or ADG of surgical castrated male growing-finishing pigs at any individual stage or over the entire trial period (*p* > 0.05; Table 2). However, during the three weeks (4–6 weeks) following the booster vaccination, FSH vaccination significantly reduced the FCR compared to the GnRH-immunized group (*p* < 0.05; Table 2). Furthermore, even when extended to the full period, although FSH vaccination did not significantly reduce FCR or increase ADG compared to the control and GnRH-immunized groups (*p* > 0.05; Table 2), it resulted in a 3.68% improvement in overall ADG and a 3.49% reduction in overall FCR relative to the control group (*p* > 0.05; Table 2), while ADFI remained largely unchanged.

### 3.3. The Impact of FSH Versus GnRH Vaccination on Carcass Traits

Compared to the placebo-immunized control group, neither GnRH nor FSH vaccination significantly affected (or “had a significant effect on”) live weight, carcass weight, or dressing percentage (*p* > 0.05; Table 3). Even though, GnRH and FSH vaccination reduced average backfat thickness by 0.90% and 4.55%, abdominal fat weight by 3.49% and 10.56%, and abdominal fat percentage by 4.09% and 11.95%, respectively, compared to the control group (Table 3). Additionally, we observed that the FSH vaccinated group exhibited a significant increase in the liver index compared to the CON group (*p* < 0.05; Table 3). Moreover, there was a tendency toward increased liver weight in the FSH group relative to the CON group (*p* = 0.054; Table 3). Thus, FSH vaccination may improve feed efficiency and growth performance of borrows by promoting liver development.

### 3.4. The Impact of FSH Versus GnRH Vaccination on Meat Quality

As shown in Table 4, compared with the control group, at slaughter FSH vaccination significantly increased muscle pH at 45 min, drip loss at 24 h, and shear force (*p* < 0.05). However, vaccination with either GnRH or FSH had no significant effect on the marbling score, meat color, cooking yield, cooking loss, moisture content, or intramuscular fat content in the longissimus dorsi muscle of finishing pigs (*p* < 0.05).

### 3.5. The Impact of FSH Vaccination on Serum Biochemical Indicators

**Figure 2:**
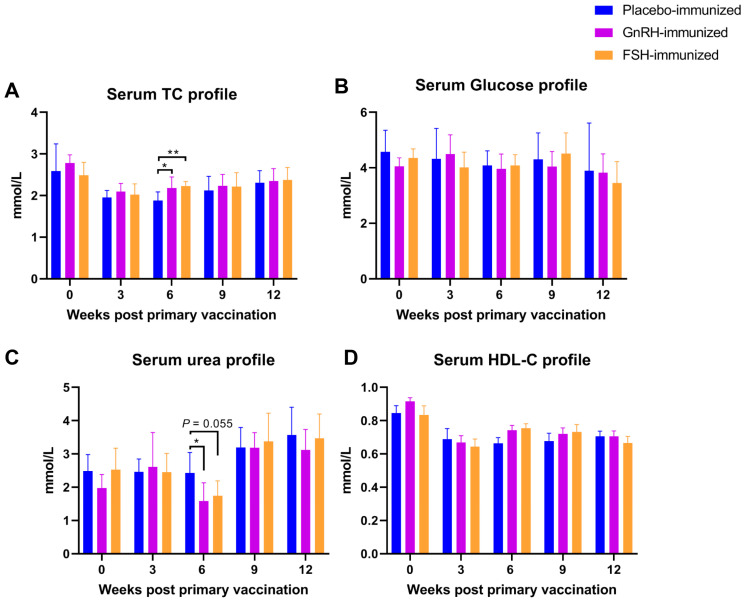
The impact of FSH versus GnRH vaccination on serum biochemical indicators. (**A**) Serum total cholesterol (TC) level; (**B**) Serum glucose level; (**C**) Serum urea level; (**D**) Serum high density lipoprotein cholesterol (HDL-C) level. * *p* < 0.05; ** *p* < 0.01.

Three weeks after the second vaccination, the serum urea level in the GnRH group was significantly lower than that in the CON group (*p* < 0.05; Figure 2); the total cholesterol (TC) levels in both the GnRH group and the FSH group were significantly higher than those in the CON group (*p* < 0.05; Figure 2). Vaccination had no significant effect on the levels of HDL-C or glucose (Glu) (*p* > 0.05; Figure 2).

## 4. Discussion

Although surgical castration of male piglets is widely practiced globally, this procedure increases carcass fat deposition and reduces feed conversion efficiency, thereby decreasing the profitability of swine production [27]. The classic explanation for increased fatness after castration is the lack of steroid hormones from the testes [3,11]. A study revealed that surgical castration significantly increases FSH levels in male pigs, which promotes fat deposition by activating the peroxisome proliferator-activated receptor gamma (PPARγ) signaling pathway in adipose tissue [16]. Consequently, modulating FSH secretion may represent a novel therapeutic target for mitigating excessive fat deposition and improving feed efficiency in castrated boars.

Both in vitro and in vivo experiments have confirmed the expression of FSHR in adipocytes, with FSH promoting lipid accumulation through receptor-mediated mechanisms in adipose tissue [13,14,15]. Research confirmed that FSH, not LH, promotes fat accumulation in gonadectomized animals. This was demonstrated by the significant increase in body weight and fat mass observed in GnRH agonist-treated, gonadectomized mice following recombinant FSH administration [13]. All these results implicate the role of FSH in regulating fat accumulation. Quantitative real-time PCR (RT-qPCR) analysis demonstrated moderate FSHR expression levels in both subcutaneous and visceral adipose tissues of castrated male pigs. And surgical castration leads to elevated serum concentrations of both FSH and LH, subsequent qPCR analysis detected no LHR expression in either adipose depot [16]. These findings were further validated by Super deepSAGE sequencing, which confirmed FSHR but not LHR expression in porcine adipose tissues [28]. These results strongly suggest that FSH, but not LH, mediates fat accumulation in male pigs.

Adipocyte FSHRs couple with Giα proteins, which inhibit cAMP-mediated β3-adrenergic receptor signaling by reducing intracellular cAMP levels [16]; this signaling pathway subsequently downregulates the expression of mitochondrial uncoupling protein 1 (UCP1) [13]. Furthermore, FSH promotes adipogenesis by upregulating key lipogenic factors, including PPARγ, CCAAT/enhancer-binding protein alpha (C/EBPα), lipoprotein lipase (LPL), and fatty acid synthase (FASN) [13,14]. In loss-of-function studies, attenuation of FSH signaling—whether in Fshr+/− mice, high-fat diet-fed mice, or ovariectomized (OVX) mice—via pharmacological intervention using FSH-neutralizing antibodies consistently upregulated UCP1 expression and promoted mitochondrial biogenesis. These effects resulted in enhanced energy expenditure and significant reductions in adipose tissue mass [13,29,30]. Therefore, FSH blockage seems to prevent adipose accumulation through simultaneously enhancing UCP1 expression and suppressing de novo adipogenesis. However, the UCP1 gene has been functionally lost in pigs during evolution [31,32]. Thus, suppressing de novo adipogenesis by blocking PPARγ signaling is likely the primary pathway through which FSH blockage reduces fat accumulation.

To evaluate a novel FSH vaccine we designed and developed, which incorporates an FSHβ13AA-tandem-ovalbumin conjugate, we first compared the effects of immunization with a GnRH vaccine, a FSH vaccine, and a placebo on the growth performance of barrows. It is worth mentioning that we applied NRC amino acid recommendations for intact boars under the hypothesis that FSH or GnRH immunocastration may attenuate adipose deposition in barrows while potentially restoring protein deposition kinetics to levels resembling those of intact males. For instance, Noblet et al. (1994) [33] reported that the digestible lysine requirement for intact boars was 0.78% of the diet, whereas castrated barrows only required 0.58%, during the 25–90 kg body weight phase. This discrepancy is attributed to boars’ enhanced lean tissue accretion efficiency and reduced lipid deposition due to androgen-mediated metabolic effects. Supporting this, Main et al. (2008) [34] determined lysine requirements of 0.91% (total basis) for 69–93 kg barrows and 1.00% for gilts in a high-lean-genetic population (n = 7801 pigs). These data suggest that intact boars likely exhibit lysine demands comparable to or exceeding those of gilts.

We found that compared to the GnRH-immunized group, the FSH vaccination group exhibited a significant improvement in feed efficiency within three weeks following booster vaccination. When evaluated over the entire 12-week period, surgically castrated boars vaccinated with the FSH vaccine showed improved feed efficiency and average daily gain compared with Placebo-immunized boars under conditions of similar average daily feed intake, although these differences were not statistically significant. We think that this may be attributed to the gradual decline in antibody levels over time, which is consistent with the observed changes in optical density values in the boars.

At 12 weeks of age (approximately 130 kg live weight), the pigs were slaughtered. The results showed that neither GnRH vaccination nor FSH vaccination significantly affected live weight, carcass weight, or dressing percentage compared to the control group. However, compared to the control, the GnRH-immunized group exhibited a 0.90% reduction in average backfat thickness and a 3.49% decrease in abdominal fat weight, while the FSH-immunized group showed more pronounced reductions of 4.55% in backfat thickness and 10.56% in abdominal fat weight. Additionally, the abdominal fat percentage decreased by 4.09% and 11.95% in the GnRH-immunized and FSH-immunized groups, respectively. These findings suggest that immunization against either GnRH or FSH can partially suppress fat deposition in castrated male pigs. The liver plays a central role in regulating metabolism, acting not only as the primary source of endogenous glucose production and the main reservoir of glycogen, but also as both a target and regulator of glucoregulatory hormones [35]. In this experiment, the FSH group showed a significant increase in the liver index compared to the CON group (*p* < 0.05). There was also a trend toward increased liver weight in the FSH group relative to the CON group, which approached statistical significance (*p* = 0.054). Therefore, we speculate that FSH immunization likely promotes liver enlargement, thereby improving the feed efficiency of barrows.

With the continuous improvement of living standards, the production of high-quality pork has become increasingly important. Pork quality is a comprehensive trait evaluated through both physical and biochemical characteristics, including meat color, pH value, water-holding capacity, intramuscular fat content, and flavor compounds [29]. Among these parameters, muscle pH and drip loss are particularly critical for meat quality assessment. A postmortem pH value at 45 min (pH 45) below 5.6 serves as a key threshold for distinguishing normal meat from abnormal meat [30]. Drip loss reflects the muscle’s water retention capacity under external forces [30]. Excessive drip loss results in pale coloration, soft texture, and exudate formation, characteristic of pale, soft, exudative (PSE) meat [30]. In this study, we investigated for the first time the effects of GnRH and FSH immunization on meat quality in surgically castrated pigs. Our results demonstrated that compared with the Placebo-immunized group, FSH vaccination significantly increased muscle pH45, 24 h drip loss, and shear force values. Postmortem pH is a fundamental meat quality parameter that reflects the rate of postmortem glycolysis (where glycogen breakdown produces lactic acid, causing pH decline). Under normal conditions, porcine muscle pH decreases rapidly from the physiological level of 7.0–7.2 to approximately 6.0–6.5 within 45 min postmortem [36]. In this trial, although the pH45 values in the FSH and GnRH groups were significantly higher compared to the control group, they remained within the acceptable range, indicating no impact on meat quality. Previous research has established that muscle tenderness positively correlates with water-holding capacity and intramuscular fat content [37], which is consistent with our findings. We observed that FSH-immunized male pigs exhibited higher shear force values and drip loss along with lower IMF content. These results indicate that active immunization against follicle-stimulating hormone leads to reduced water-holding capacity and tenderness in muscle tissue [17]. The underlying mechanisms warrant further investigation through hormone assays, muscle metabolomics, and related analyses.

From the animal welfare perspective, immunocastration eliminates the risks of surgical pain, hemorrhage, and wound infections associated with physical castration [2,10]. But surgical castration remains the predominant practice in global swine production, particularly in China, where economic factors such as production costs heavily influence management decisions. From an endocrinological perspective, optimal production performance requires careful balancing of testicular steroids and gonadotropin levels. Specifically, adequate testosterone concentrations should be maintained during the early fattening phase to capitalize on its anabolic effects; subsequent significant reduction in FSH activity is necessary to minimize excessive fat deposition. GnRH plays a central role in the regulation of reproductive functions and behaviors in animals via the hypothalamic-pituitary-gonadal (HPG) axis, serving as a key neurohormone for both the initiation and modulation of reproductive physiology [38]. It is released into the bloodstream in a pulsatile manner and subsequently binds to GnRH receptors (GnRH-R) in the pituitary gland, stimulating the synthesis and secretion of LH and FSH [37].

In this study, compared with the GnRH group, the FSH group exhibited a significant improvement in feed efficiency only at 3 weeks post-booster vaccination, with no significant differences observed between the two treatments during the remaining phases. We speculate that this may be attributed to the fact that GnRH immunization not only reduces FSH concentrations but also suppresses LH levels [39,40,41], and LH concentration may also exert a certain influence on feed efficiency in surgically castrated male growing-finishing pigs. But further validations are required.

The potential implementation of FSH immunization as a widespread practice in the swine industry necessitates careful consideration of its practical and economic impacts. Firstly, the cost of the vaccine itself will be a primary determinant of its adoption. While this study demonstrates promising efficacy, the commercial viability will depend on whether the production cost can be kept sufficiently low to offer a compelling return on investment for producers. Currently, the cost of two doses of this newly developed FSH vaccine is approximately USD 2.2. Further optimizations will be made to both the vaccine formulation and its associated costs. Secondly, the current protocol requiring two hormone injections poses significant challenges in terms of animal handling. This doubles the labor requirements and increases stress for both the animals and the handlers, which could limit its application in large-scale operations. Efficient handling facilities and streamlined protocols would be essential to mitigate this drawback. Finally, producers must weigh the trade-off between the economic benefits and potential changes in carcass quality. Our findings indicate that FSH immunization improves lean meat yield and feed efficiency, which is economically advantageous. However, any potential, albeit unobserved in this study, negative impact on other quality attributes must be thoroughly evaluated. The overall economic benefit will thus be a function of the premium for superior carcass leanness against the combined costs of the vaccine, additional labor, and any potential discounts for altered quality. Future studies incorporating a full cost–benefit analysis will be crucial to conclusively determine the commercial feasibility of this immunocastration strategy.

In a nutshell, we firstly demonstrated that FSH vaccination could effectively improve feed efficiency and reduce fat deposition in castrated boars. Although the practical impact of FCR improvement was only observed at 4–6 weeks, the overall FCR of the FSH group was still 3.49% higher than that of the control group. Similarly, while no significant differences in fat deposition were detected between treatments, the carcass dressing percentage of the FSH group was 0.28% greater than that of the control, contributing to certain economic benefits. We think this may tightly be related to the antibody titers, which were transiently high during 4–6 weeks of treatment, and then declined. This was possibly the reason for why there was no significant difference in fat deposition between placebo-immunized and FSH-immunized male pigs. All in all, immunoneutralizing FSH activities may represent a novel therapeutic target for mitigating excessive fat deposition and improving feed efficiency in castrated boars.

## 5. Conclusions

In summary, although compromising carcass meat quality attributes, our results suggest that FSH vaccination could efficiently improve feed efficiency and reduce adiposity in barrows. Additionally, FSH vaccination resulted in a notable increase in liver weight, suggesting potential metabolic alterations. These findings highlight the promising role of FSH vaccination as a potential strategy to improve production efficiency and body composition in swine production. However, the mechanism by which FSH vaccination improves feed efficiency and body composition of barrows requires further investigation. Furthermore, the optimal immunization protocol for FSH vaccine-induced feed efficiency improvement in barrows needs additional research and refinement.

## Figures and Tables

**Table 1:** Dietary formulations (as fed basis).

Ingredients	Composition, %		
	25–50 kg	50–75 kg	75–130 kg
Corn	70.80	73.04	72.16
Soybean Meal, CP 44%	19.10	15.12	15.10
Wheat Bran	5.05	7.00	8.25
Soybean oil	1.90	1.80	1.70
Dicalcium Phosphate	0.91	0.88	0.80
Limestone	0.87	0.81	0.81
Feed-grade Sodium Chloride	0.30	0.35	0.35
Choline Chloride, 50%	0.10	0.15	0.15
L-Lysine Hydrochloride, 98.5%	0.54	0.49	0.40
DL-Methionine, 99%	0.08	0.05	0.03
L-Threonine, 98.5%	0.18	0.16	0.11
L-Tryptophan, 98%	0.04	0.02	0.01
Mineral Premix ^1^	0.10	0.10	0.10
Vitamin Premix ^2^	0.03	0.03	0.03
Total, %	100	100	100

^1^ Mineral premixes provided the following per kg of diets: Fe (FeSO_4_), 60 mg; Cu (CuSO_4_), 4 mg; Zn (ZnSO_4_), 60 mg; Mn (MnSO_4_), 2 mg; I (KI), 0.14 mg; Se (Na_2_SeO_3_), 0.2 mg, provided by Kunming Saturn Biological Technology (Group) Co., Ltd. (Kunming, China). ^2^ The composition of vitamin premix: Vitamin A 24,000,000 IU, Vitamin D_3_ 4,800,000 IU, Vitamin E 200,000 IU, Vitamin K_3_ 9600 mg, Vitamin B_1_ 4000 mg, Vitamin B_2_ 14,400 mg, Vitamin B_6_ 7200 mg, Vitamin B_12_ 50 mg, Niacinamide 80,000 mg, Folic Acid 8000 mg, D-Biotin 960 mg, D-Pantothenic Acid 50,000 mg, provided by Kunming Saturn Biological Technology (Group) Co., Ltd. (Kunming, China).

**Table 2:** Effects of FSH versus GnRH vaccination on growth performance of finishing pigs.

Items	Placebo-Immunized	GnRH-Immunized	FSH-Immunized	*p*-Value
				ANOVA
Sample Size, n	8	8	8	
1–3 wk				
ADFI, kg/d	1.85 ± 0.08	1.92 ± 0.04	1.85 ± 0.08	0.121
ADG, g/d	1047.66 ± 48.53	1045.05 ± 70.07	1018.75 ± 52.28	0.551
FCR	1.77 ± 0.10	1.84 ± 0.13	1.82 ± 0.05	0.343
4–6 wk				
ADFI, kg/d	2.43 ± 0.07	2.45 ± 0.05	2.42 ± 0.08	0.590
ADG, g/d	1121.32 ± 51.99	1112.13 ± 69.40	1165.44 ± 66.12	0.218
FCR	2.17 ± 0.11 ^ab^	2.21 ± 0.16 ^a^	2.08 ± 0.11 ^b^	0.128
7–9 wk				
ADFI, kg/d	3.44 ± 0.21	3.52 ± 0.13	3.48 ± 0.10	0.643
ADG, g/d	1395.83 ± 134.05	1434.52 ± 87.02	1482.14 ± 135.74	0.378
FCR	2.48 ± 0.16	2.46 ± 0.12	2.36 ± 0.19	0.332
10–12 wk				
ADFI, kg/d	3.10 ± 0.05	3.09 ± 0.05	3.11 ± 0.07	0.796
ADG, g/d	724.61 ± 114.19	681.64 ± 126.09	777.35 ± 164.46	0.390
FCR	4.38 ± 0.74	4.70 ± 1.11	4.16 ± 0.90	0.551
Overall				
ADFI, kg/d	2.71 ± 0.07	2.74 ± 0.04	2.72 ± 0.06	0.529
ADG, g/d	1017.96 ± 71.18	1009.74 ± 24.79	1055.39 ± 71.13	0.288
FCR	2.67 ± 0.16	2.71 ± 0.05	2.58 ± 0.17	0.191

^a, b^ Different letters denote statistical significance at *p* < 0.05.

**Table 3:** Effects of FSH versus GnRH vaccination on slaughter performance of finishing pigs.

Items	Placebo-Immunized	GnRH-Immunized	FSH-Immunized	*p*-Value
Sample Size, n	6	6	6	
Average body weight, kg	131.50 ± 6.80	129.67 ± 3.01	131.67 ± 6.22	0.793
Carcass weight, kg	100.65 ± 4.40	100.13 ± 2.46	101.17 ± 4.11	0.893
Dressing percentage, %	76.58 ± 1.35	77.24 ± 1.71	76.86 ± 0.81	0.697
Average Backfat, mm	22.52 ± 2.69	22.32 ± 2.12	21.54 ± 2.82	0.785
Ham and hip weight, kg	16.42 ± 0.60	16.53 ± 1.31	17.73 ± 2.57	0.362
Ham and hip rate, %	33.12 ± 1.14	33.00 ± 2.10	35.17 ± 4.17	0.341
Leaf fat weight, kg	1.78 ± 0.45	1.72 ± 0.49	1.61 ± 0.31	0.775
Leaf fat rate, %	1.78 ± 0.49	1.71 ± 0.46	1.59 ± 0.27	0.722
Right hemisomal weight, kg	49.49 ± 2.40	50.17 ± 1.26	50.10 ± 1.90	0.798
Head weight, kg	7.91 ± 0.32	7.43 ± 0.33	7.73 ± 0.74	0.274
Hoof weight, kg	2.13 ± 0.08 ^a^	1.93 ± 0.12 ^b^	2.03 ± 0.18 ^ab^	0.061
Gut weight, kg	10.35 ± 1.40	9.71 ± 0.51	10.78 ± 1.97	0.442
Longissimus dorsi weight, kg	3.40 ± 0.35	3.38 ± 0.22	3.26 ± 0.29	0.708
Psoas major weight, kg	0.48 ± 0.06	0.45 ± 0.04	0.44 ± 0.01	0.295
Ribeye area, cm^2^	75.65 ± 19.45	62.08 ± 8.50	66.66 ± 8.63	0.228
Heart weight, kg	0.45 ± 0.03	0.43 ± 0.03	0.47 ± 0.05	0.288
Heart rate, %	0.44 ± 0.04	0.43 ± 0.03	0.46 ± 0.05	0.391
Liver weight, kg	1.72 ± 0.10	1.78 ± 0.11	1.87 ± 0.12	0.683
Liver rate, %	1.30 ± 0.02 ^a^	1.38 ± 0.01 ^ab^	1.42 ± 0.01 ^b^	0.669
Splenic organ weight, kg	0.18 ± 0.02	0.17 ± 0.02	0.17 ± 0.01	0.518
Splenic organ rate, %	0.18 ± 0.02	0.17 ± 0.03	0.17 ± 0.01	0.613
Lung weight, kg	0.79 ± 0.29	0.79 ± 0.21	0.77 ± 0.17	0.982
Lung rate, %	0.78 ± 0.26	0.79 ± 0.23	0.76 ± 0.18	0.974
Kidney weight, kg	0.31 ± 0.03	0.31 ± 0.01	0.33 ± 0.02	0.169
Kidney rate, %	0.30 ± 0.03	0.31 ± 0.02	0.33 ± 0.02	0.152

^a, b^ Different letters denote statistical significance at *p* < 0.05.

**Table 4:** Effects of FSH versus GnRH vaccination on carcass traits of finishing pigs.

Items	Placebo-Immunized	GnRH-Immunized	FSH-Immunized	*p*-Value
				ANOVA
Sample Size, n	6	6	6	
Marble pattern score	1.83 ± 0.41	1.67 ± 0.52	1.50 ± 0.55	0.521
45 min L* (%)	82.14 ± 9.13	83.07 ± 4.54	86.30 ± 4.21	0.510
24 H L* (%)	55.18 ± 1.84	54.90 ± 2.34	57.67 ± 5.32	0.348
24 H a*	11.03 ± 0.68	10.58 ± 0.15	10.49 ± 1.02	0.634
24 H b*	5.95 ± 0.80	6.71 ± 0.02	6.51 ± 0.36	0.239
48 H L* (%)	51.43 ± 1.46	54.28 ± 2.29	52.62 ± 2.19	0.294
48 H a*	10.09 ± 0.53	10.09 ± 0.39	10.81 ± 1.12	0.443
48 H b*	5.54 ± 0.30	5.71 ± 0.52	6.04 ± 0.29	0.345
45 min pH	6.02 ± 0.39 ^a^	6.37 ± 0.28 ^ab^	6.46 ± 0.37 ^b^	0.106
24 h pH	5.27 ± 0.08	5.27 ± 0.05	5.25 ± 0.08	0.953
48 h pH	5.33 ± 0.05	5.32 ± 0.07	5.39 ± 0.08	0.401
Drip loss 24 h (%)	1.82 ± 0.42 ^a^	2.66 ± 0.83 ^ab^	3.09 ± 0.75 ^b^	0.019
Drip loss 48 h (%)	4.65 ± 0.99	4.92 ± 0.50	4.18 ± 1.50	0.505
Cooked meat rate (%)	68.35 ± 2.33	67.09 ± 2.81	68.72 ± 2.73	0.545
Cooking loss (%)	31.65 ± 2.33	32.91 ± 2.81	31.28 ± 2.73	0.545
Shear force (Kg)	9.23 ± 2.13 ^a^	9.67 ± 2.41 ^ab^	12.87 ± 3.59 ^b^	0.077
Moisture (%)	72.04 ± 1.05	71.33 ± 1.43	72.00 ± 1.19	0.548
Intramuscular fat (%)	2.50 ± 0.64	2.54 ± 0.58	2.00 ± 0.91	0.385

^a, b^ Different letters denote statistical significance at *p* < 0.05.

## Data Availability

The data provided in this study are available upon request from the corresponding author.

## Abbreviations

The following abbreviations are used in this manuscript:

FSH	Follicle-stimulating hormone
GnRH	Gonadotropin-Releasing Hormone
LH	Luteinizing Hormone
ADG	Average daily gain
ADFI	Average daily feed intake
FCR	Feed conversion ratio
L*	Lightness
a*	Red-Green Chromaticity Component
b*	Blue-Yellow Chromaticity Component
TC	Total cholesterol
Glu	Glucose
HDL-C	High density lipoprotein cholesterol
TSH	Thyroid-stimulating hormone
UCP1	Uncoupling protein 1
C/EPBα	CCAAT/enhancer-binding protein alpha
LPL	Lipoprotein lipase
FASN	Fatty acid synthase
